# Alexithymia, Dissociation, and Family Functioning in a Sample of Online Gamblers: A Moderated Mediation Study

**DOI:** 10.3390/ijerph182413291

**Published:** 2021-12-16

**Authors:** Eleonora Topino, Alessio Gori, Marco Cacioppo

**Affiliations:** 1 Department of Human Sciences, LUMSA University of Rome, Via della Traspontina 21, 00193 Rome, Italy; m.cacioppo@lumsa.it; 2 Department of Health Sciences, University of Florence, Via di San Salvi 12, Pad. 26, 50135 Florence, Italy; alessio.gori@unifi.it

**Keywords:** pathological gambling, internet gambling, online gambling, risk factors, protective factors, moderated mediation analysis

## Abstract

The diffusion of the internet and technological progress have made gambling on online platforms possible, also making it more anonymous, convenient, and available, increasing the risk of pathological outcomes for vulnerable individuals. Given this context, the present study explores the role of some protective and risk factors for problematic gambling in online gamblers by focusing on the interaction between alexithymia, dissociation, and family functioning. A sample of 193 online gamblers (M_age_ = 28.8 years, SD = 10.59; 17% females, 83% males) completed the South Oaks Gambling Screen, Twenty-Items Toronto Alexithymia Scale, Dissociative Experience Scale-II, and Family Adaptability and Cohesion Evaluation Scales-IV through an online survey. MANOVA, ANOVA and moderated mediation analyses were carried out to analyse the data. Significant differences in cohesive family functioning, alexithymia and dissociation have been found between online gamblers with problematic, at-risk or absent levels of gambling disease. Furthermore, the results showed a significant and positive association between alexithymia and problematic online gambling, partially mediated by dissociation, with the moderation of cohesive family functioning. Such data may have relevant clinical implications, highlighting the interaction of some core personal and environmental variables that may be involved in the etiology of online pathological gambling and could be kept in mind to tailor preventive interventions.

## 1. Introduction

Although the diffusion of the internet improved many aspects of everyday life through a greater speed and availability of services, it may also be a way of accessing activities with potentially psychopathological outcomes, such as online gambling [1]. Gambling disorder is a behavioural addiction characterized by a high and pervasive involvement in gambling activities despite significant adverse consequences and significant impairment of the subject’s functioning in different areas of life [2]. It has been included within the category of “Substance-Related and Addictive Disorders” in the fifth edition of the *Diagnostic and Statistical Manual of Mental Disorders* (DSM-5), given the numerous vulnerability factors, neurobiological correlates, and psychopathological symptoms shared with substance use addiction [2,3]. Technological progress has made gambling practicable on online platforms, providing the characteristics of availability, ease of access, confidentiality, and anonymity to this activity [4]. However, given the relationship between the availability of gambling opportunities and increasing levels of related problems [5], previous evidence has suggested that online practice is more associated with the severity of gambling disorder than land-based practice [1,6]. As the online gambling environment is increasingly widespread and appears to represent a significantly greater risk for vulnerable gamblers [7], the study of the key factors related to the psychopathological drift of this activity has acquired growing attention in the scientific literature (see Kuss and Griffiths [6] for a review).

In this regard, the scientific literature has highlighted the need to analyse the core elements for the development and maintenance of addictive behaviours, considering both individual and contextual aspects (e.g., [8,9,10]). These levels interact with each other in contributing to vulnerability to problematic gambling, and it is, therefore, important to consider them not in an isolated way, but in light of their mutual influence [11]. Given this framework, the present study offers an analysis of some protective and risk factors for problematic gambling in online gamblers, by focusing on the interaction of some personal (i.e., alexithymia and dissociation) and environmental (i.e., family functioning) variables.

Alexithymia is a form of emotional dysregulation characterized by difficulties in identifying and describing emotions, as well as in understanding their physiological correlates, with attention focused outwardly [12]. It has been frequently indicated as a key transdiagnostic factor in the etiology of several psychological and addictive disorders [9,13], including gambling disorder [14,15], which has been associated with altered emotional processes [16]. In this regard, the theory of self-medication postulated that addictive behaviours may be a way of alleviating negative affective states [17]. This theory has been further investigated and applied in subsequent research, which highlighted that pathological gambling may become an external compensatory strategy of emotional regulation, used to escape dysregulated internal emotional states [18,19]. In confirmation of this, Marchetti, Verrocchio and Porcelli recently elaborated a systematic literature review [20], where they found higher alexithymic features in subjects with pathological scores of gambling behaviour, both at the community and clinical levels, further supporting the hypothesis that alexithymia may be a central factor in pathological gambling, which becomes, itself, a strategy to avoid negative emotions.

As highlighted by Gori and colleagues [15] in their Comprehensive Model for Gambling Behaviors, dissociation appears as another core personal variable for vulnerability to pathological gambling. Indeed, dissociation was significantly associated with alexithymia [15] and has been found to be associated with levels of problematic gambling [14,21,22,23]. More specifically, it can be experimented with on a continuum involving adaptive and common forms of absorption, on one side, to detached modes that are dysfunctional and pathological, on the other [24,25]. Concerning addiction, dissociation may be linked with the motivation to escape from negative emotional experiences [26,27]; therefore, pathological gambling can be linked to states of evasion in response to broad emotional vulnerability [23]. In other words, gambling behaviour could be considered a dissociative phenomenon aimed at moving away from painful mental states [28], and could, therefore, be initially used as a defence that, however, may lead to a condition of constant absorption and facilitating the onset and maintenance of addiction [9,14].

Among the environmental variables, family functioning, defined as the overall quality of family life [29], appears as a particularly promising variable that may have a significant influence on the development of addiction (e.g., [30]). In this regard, an important reference model is the Circumplex Model of Marital and Family Systems, by Olson and colleagues [29], which systematize family functioning by considering the levels of cohesion (i.e., the emotional bonding among the family members), flexibility (i.e., the quality of family organization, role relationship, as well as rules and negotiations), and communication (i.e., the positive communication skills utilized in the family system), dimensions that have frequently been associated with mental health [31,32]. Indeed, family members have a profound effect on mutual functioning and well-being and can play an important role in the development of gambling [33]. Family members may recognize the first signs of gambling problems and stimulate a reaction to them [34]. Parallelly, previous research showed that people with gambling disorder display unhealthy and unbalanced family functioning (see [35,36] for reviews).

Taking these aforementioned findings into account, the present study aims at:

- exploring the differences in the analysed variables, based on gambling severity in online regular gamblers, to provide a better understanding of the characteristics related to problematic online gambling; and
- investigating the relationship between alexithymia, dissociation, and family functioning in contributing to problem gambling among online gamblers.

Given the existing scientific literature, we expected to find significantly higher levels of alexithymia and dissociation, as well as less family functioning in online gamblers with higher levels of problem gambling. Furthermore, based on these differences, a moderated-mediation model is hypothesized, in which: (i) alexithymia is associated with problematic online gambling; (ii) dissociation mediates the relationship between alexithymia and problematic online gambling; (iii) adaptive and/or maladaptive family functioning styles moderate the relationship between dissociation and problematic online gambling.

## 2. Materials and Methods

### 2.1. Participants, Procedure and Ethics

The sample comprised 193 regular gamblers who declared to engage in gambling behaviours mainly online. Their mean age was of 28.8 years (*SD* = 10.59; range 20–78 years) and they were pronominally men (82.9%), single (78.8%), employed (37.8%), and have a high school diploma (54.9%), as reported in Table 1. All participants were recruited online through a snowball-like procedure, by sending out an anonymous link to the survey on online websites and discussion forums on various types of gambling activities. The administration of the questionnaires, together with a demographic questionnaire, was implemented on the *Google Forms* platform and it took about 20 min to complete. Before starting the survey, all the respondents were informed about the general aim of the research and provided informed consent electronically. Involvement in the study was voluntary and each participant was free to stop filling out the survey and leave the research at any moment. All the procedures performed in the study involving human participants have been approved by the Ethics Committee for Scientific Research (CERS) of the LUMSA University of Rome, Italy.

### 2.2. Measures

#### 2.2.1. South Oaks Gambling Screen (SOGS)

The *South Oaks Gambling Screen* (SOGS; Lesieur and Blume [37]; Italian version: Guerreschi and Gander [38]) is a 16-item self-report instrument for the screening of the presence and severity of pathological gambling (PG). Items (e.g., “*When you gamble, how often do you go back another day to win back money you lost?*”) have different response formats: some are on a three-point scale option (“*not at all*”; “*Less than once a week*”; “*Once a week or more*”), others are multiple-choice, still others are yes/no items. The SOGS allows to classify participants into three groups: absence of gambling disease (zero-to-two scores); at risk for gambling disease (three-to-four scores), and problematic gambling (scores of five or more). In this study, the Italian version was used and showed excellent internal consistency in the present sample (the Cronbach’s α value is 0.92).

#### 2.2.2. Twenty-Items Toronto Alexithymia Scale (TAS-20)

The *Twenty-Items Toronto Alexithymia Scale* (TAS-20; Bagby, Parker and Taylor [39]; Bagby, Taylor and Parker [40]; Italian version: Bressi et al. [41]) is a 20-item self-report instrument for the evaluation of the level of alexithymia. Items were on a five-point Likert scale (from one = “*strongly disagree*” to five = “st*rongly agree*”), allowing for both a total score and three subscales: difficulty identifying feelings (e.g., “*I am often confused about what emotion I am feeling*”); difficulty describing feelings (e.g., “*It is difficult for me to find the right words for my feelings*”); externally oriented thinking (e.g., “*I prefer to analyse problems rather than just describe them*”). The total score of the TAS-20 allows classifying participants into three groups: a cut off above a value of 61 indicates an alexithymic condition; scores equal to or less than 51 indicate no alexithymia; scores between 52 and 60 detect a possibility of alexithymia. In this study, the total score of the Italian version was used, which showed good internal consistency in the present sample (the Cronbach’s α value is 0.82).

#### 2.2.3. Dissociative Experience Scale-II (DES-II)

The *Dissociative Experiences Scale-II* (DES-II; Carlson and Putnam [42]; Italian version: Schimmenti [43]) is a 28-item self-report instrument for evaluating levels of dissociative experiences by considering a variety of dissociation types. Items were on an 11-point scale (from 0% = “*never*,” to 100% = “*always*”), allowing for both a total score and three subscales: dissociative amnesia (e.g., “*Some people have the experience of finding themselves in a place and have no idea how they got there. Circle the number to show what percentage of the time this happens to you*”); absorption (e.g., “*Some people find that sometimes they are listening to someone talk and they suddenly realize that they did not hear part or all of what was said. Circle the number to show what percentage of the time this happens to you”*); depersonalization-derealization (e.g., “*Some people have the experience of looking in a mirror and not recognizing themselves. Circle the number to show what percentage of the time this happens to you*”). Higher scores indicate greater levels of psychological dissociation. In this study, the total score of the Italian version was used, which showed excellent internal consistency in the present sample (the Cronbach’s α value is 0.95).

#### 2.2.4. Family Adaptability and Cohesion Evaluation Scales-IV (FACES IV)

The *Family Adaptability and Cohesion Evaluation Scales-IV* (FACES IV; Olson [44]; Italian version: Baiocco et al., [45]) is a 42-item self-report instrument for the evaluation of family functioning, following the theoretical guide of the Circumplex Model of Marital and Family Systems [29]. Items are scored on a five-point Likert scale (from one = “*Strongly Disagree*”, to five “*Strongly Agree*”) and allow for six scales, of which the first two indicate balanced functioning, while the other four unbalanced functioning: cohesion (e.g., “*Family members are involved in each others lives*”), flexibility (e.g., “*Our family tries new ways of dealing with problems*”), disengaged (e.g., “*We get along better with people outside our family than inside*”), enmeshed (e.g., “*We spend too much time together*), rigid (e.g., “*There are strict consequences for breaking the rules in our family*”), chaotic (e.g., “*We never seem to get organized in our family*”). Higher scores indicate greater levels of the family functioning indicated in the specific subscale. In the present study the Italian version was used, for which the six scales showed good internal consistency in the present sample (cohesion, α = 0.89; flexibility, α = 0.84; enmeshed, α = 0.75; disengaged, α = 0.78; chaotic, α = 0.67; rigid α = 0.73).

### 2.3. Data Analysis

The collected data were analysed with the Statistical Package for the Social Sciences (SPSS) version 21.0 (IBM, Armonk, NY, USA) for Windows. Since the link to the survey has been distributed to the Internet through a snowball-like procedure and participation was voluntary, anonymous and without any formal registration, it was not possible to calculate the response rate. Furthermore, there was no missing data in the data response set because the online platform used did not allow the submission of surveys unless all items were answered. The significance threshold was arbitrarily chosen at 0.05 unless otherwise specified. Descriptive statistics were calculated. Relationships between variables were computed through a Pearson’s correlation analysis. The multivariate analysis of variance (MANOVA) was performed to compare the types of Family functioning (dependent variables) based on the levels of gambling disease, by inserting the SOGS groups (absence of gambling disease, At Risk for Gambling Disease, problematic gambling) as the independent variable. Separate analyses of variance (ANOVAs) were conducted to support the interpretation in mean scores by setting a Bonferroni-adjusted *p*-value of 0.008 as the criterion of significance, also implementing post-hoc analyses using a Scheffé test. Moreover, a series of analyses of variance (ANOVAs) was used to explore differences in alexithymia and dissociation (dependent variables) between the SOGS groups (independent variable), with Scheffé tests for post-hoc analyses. Then, the hypothesized moderated mediation was tested by performing model 4 in the macro program PROCESS 3.4 [46]. The 95% confidence interval (CI) was calculated for each regression coefficient. The statistical stability of the model was probed by following the Wayne et al., [47] technique and by performing the bootstrapping procedures with a 95% confidence interval (CI) at 5000 samples. The first allowed for an investigation of the conditional effect at the three different levels (−1DS, Mean, +1DS) of the moderators, while the bootstrap approach indicates the significance of effect when the CI (from lower limit confidence interval [Boot LLCI] to upper limit confidence interval [Boot ULCI]) does not include zero.

## 3. Results

Based on the SOGS cut-off, 57% of the participants reported an absence of gambling disease (N = 110), 17% could be considered at risk for gambling disease (N = 33), 26% described their problematic gambling (N = 50) (see Table 2). In Table 2, the correlations, means and standard deviations of the variables are shown.

The correlational analysis (see Table 2) highlighted significant and positive associations between the levels of problematic gambling and dissociation (*r* = 0.330, *p* < 0.01), alexithymia (*r* = 0.284, *p* < 0.01), and enmeshed family functioning (*r* = 0.180, *p* < 0.05), while a significant and negative relationship was shown with cohesion (*r* = −0.241, *p* < 0.01).

Results of the MANOVA highlighted a statistically significant difference in family functioning based on the levels of gambling disease, *F* (12, 370) = 2.603, *p* < 0.01; Wilk’s Λ = 0.850, partial *η^2^* = 0.078. Specifically, a significant lower level of cohesion in problematic gamblers (*M* = 20.540, *SD* = 6.801), than in those at risk (*M* = 21.000, *SD* = 6.955) or with SOGS scores indicating an absence of gambling disease (*M* = 24.227, *SD* = 6.010), as resulting from the separate follow up ANOVAs and Scheffé test: *F* (2,190) = 7.171, *p* < 0.001 (see Table 3).

Furthermore, significant higher levels of alexithymia were shown in problematic gamblers (*M* = 55.220, *SD* = 10.725), than in those at risk (*M* = 47.182, *SD* = 10.463), or with SOGS scores indicating an absence of gambling disease (*M* = 47.036, *SD* = 10.932): *F* (2, 190) = 10.549, *p* < 0.001 (see Table 4). Concerning dissociation, significantly higher scores were found in problematic gamblers (*M* = 41.223, *SD* = 17.242), than in those with absence of gambling disease (*M* = 30.497, *SD =* 13.916): *F* (2, 190) = 8.935, *p* < 0.001 (see Table 4).

**Figure 1:**
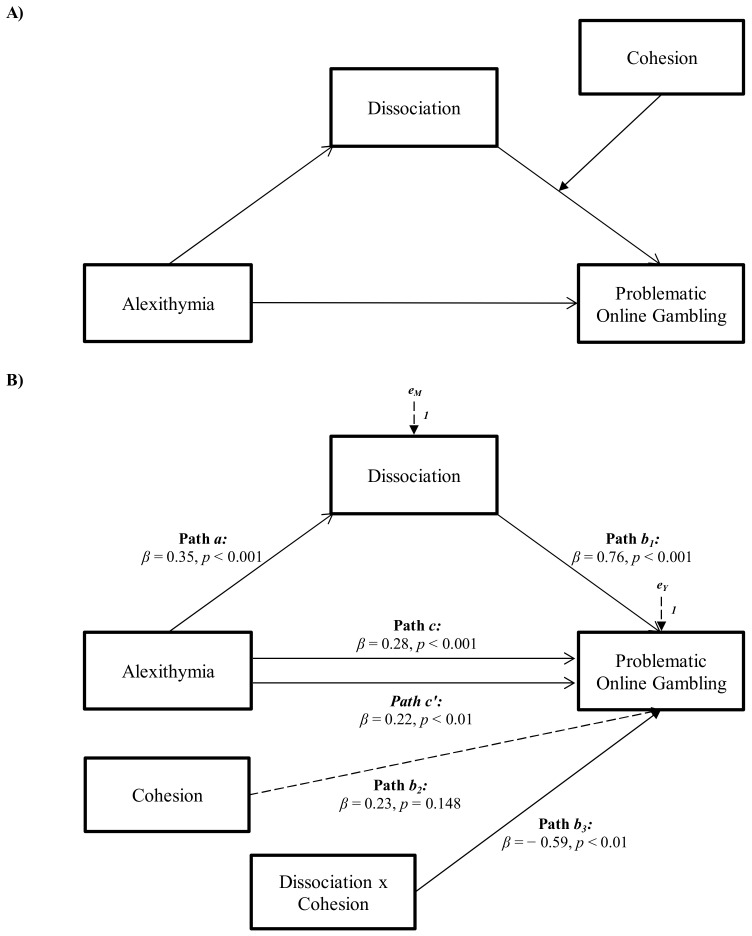
Statistical (**A**) and conceptual (**B**) forms of the moderated mediation model: the mediation of dissociation in the relationship between alexithymia and problematic gambling and the moderation of cohesion.

The moderated mediation analysis revealed that dissociation partially mediated the relationship between alexithymia and problematic gambling, and the relationship between dissociation and problematic gambling was moderated by cohesion (see Figure 1).

Specifically, alexithymia showed a significant and positive total effect on problematic gambling (Path *c* in Figure 1B; *β* = 0.28, *p* < 0.001, LLCI = 0.0549–ULCI = 0.1568). It was also significantly and positively associated with dissociation, the mediator variable (Path *a* in Figure 1B; *β* = 0.35, *p* < 0.001, LLCI = 0.2971–ULCI = 0.6625), which, in turn, was significantly and positively related with problematic gambling (path *b_1_* in Figure 1B; *β* = 0.76, *p* < 0.001, LLCI = 0.0886–ULCI = 0.0324). Furthermore, the effect of the mediator on problematic gambling was found to significantly influenced by the effect of cohesion, the moderating variable (path *b_3_* in Figure 1B; *β* = −0.59, *p <* 0.01, LLCI = −0.1277–ULCI = −0.0018), and the index of moderated mediation was found to be significant (index = −0.0035, boot LLCI = −0.0070–boot ULCI = −0.0004): Δ*R^2^* = 0.030, *F* (1, 188) = 6.861, *p* < 0.01. When included in the model, the moderated effect of dissociation partially mediated the effect of alexithymia on problematic gambling, reducing the direct effect, which however remained significant (path *c*′ in Figure 1B; *β* = 0.22, *p* < 0.01, LLCI = 0.0306–ULCI = 0.1356): *R^2^* = 0.190 *F* (4, 188) = 10.987, *p* < 0.001 (see Table 5).

**Figure 2:**
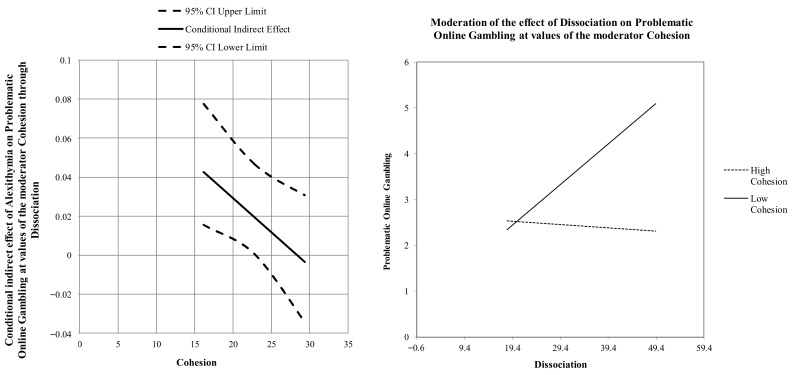
Graphic representation of the moderated-moderation effect.

Furthermore, the statistical stability of the model was further investigated by testing the conditional effects of dissociation at three levels of cohesion (i.e., −1SD, mean, +1SD). The association between dissociation and problematic gambling was stronger at low levels of cohesion (estimate = 0.089 [0.02], *p* < 0.001, LLCI = 0.0434–ULCI = 0.1340) and became non-significant at average (estimate = 0.041 [0.02], *p* = 0.056, LLCI= −0.0011–ULCI = 0.0825) and at high levels (estimate = −0.0073 [0.03], *p* = 0.822, LLCI = −0.0710–ULCI = 0.0564). Therefore, when participants reported higher levels of family cohesion, the positive indirect effect of alexithymia on problematic gambling via dissociation weakened to become non-significant (see Figure 2). 

Finally, the bootstrap analysis confirmed the statistical relevance and robustness of the moderation effect: Boot LLCI = −0.0134–Boot ULCI = −0.0008.

## 4. Discussion

Technological and digital advances have offered new gambling opportunities, making it more available, accessible, convenient, and anonymous in its practice on online platforms [1,4]. Since previous evidence points to a greater risk of addiction for this modality [1,6] and problematic online gambling appears to be associated with lower levels of mental health [48], the present research aimed to explore some risk and protective factors for the levels of problematic gambling, considering the interaction between some personal (i.e., alexithymia and dissociation) and environmental (i.e., Family functioning) variables. 

The first hypothesis was addressed by finding differences concerning the personal and environmental variables considered between online gamblers with problematic, at-risk or absent levels of gambling disease. As a result of the performed analyses, significant differences were identified in the levels of cohesive family functioning, alexithymia, and dissociation. 

With regard to family functioning, significantly lower levels of cohesion were shown in subjects with problematic online gambling, compared with the group of gamblers at risk of or with no disorder, which is consistent with previous studies focused on the relationship between family support and land-based gambling [34,49,50]. These data corroborate the role of family functioning in influencing mental health and well-being (e.g., [51,52], as well as its effect in behavioural addiction, as suggested in the evidence from problematic internet use [53], smartphone addiction [54] and internet gaming disorder [55], among others.

Furthermore, significantly higher levels of alexithymia were highlighted in subjects with problematic online gambling, compared to the group of gamblers at risk or with an absence of disorder, in line with previous studies focused on land-based gambling [56,57]. This is consistent with evidence showing that alexithymia was negatively related to resilience and well-being [58,59], and had a significant role in the etiology of substance addictions (e.g., [60,61] or other behavioural addictions, such as internet addiction [62], compulsive buying [63], or exercise addiction [64]. Additionally, the group of online gamers with problematic SOGS scores reported average alexithymia above the cut-off of 51, indicating risk levels. This further corroborates the hypothesis that addictive behaviours may arise as an attempt by alexithymic individuals to self-regulate their emotions [20], suggesting that higher levels of alexithymia may increase the risk for loss of control in online gambling.

Concerning dissociation, significantly higher levels were found in subjects with problematic online gambling, compared to the group of gamblers with an absence of disorder, echoing previous evidence on land-based gambling [21,28]. This is in line with existing research focusing on the negative effects of high levels of dissociation on mental health (see Lyssenko et al., [65] for a meta-analysis), and showed significant associations with other addictions, such as sexual addiction [66] or internet addiction [67], to name a few. Furthermore, there is an open debate in the scientific literature (see Rogier et al., [22] for a review) regarding the possibilities that dissociation may be a risk factor for the onset of gambling disorder or a consequence of it (e.g., [21,68]). Although this variable may be an element stimulated by gambling [68], in the present study, the increase in means in dissociation scores obtained as the severity of the SOGS scores increased, as well as the significant differences between problematic online gamblers and the at-risk or no-risk groups, supports the hypothesis that dissociation may also play a role in the etiology of the disorder. Longitudinal studies may further clarify this hypothesis, which is further followed in the moderated mediation model implemented in the present research. 

The results support the hypothesized model and confirm the influence of alexithymia in problematic online gambling, both directly and indirectly, taking into account the partial mediation of dissociation and the moderation of cohesive family functioning. In other words, deficits in emotional processing and regulation may prevent gamblers from managing and containing internal distress, resulting in attempts at external regulation [18,69]. In mediating this relationship, dissociation can be seen as an extreme way to cope with dysregulated negative effects, further impeding their processing and awareness, thus fuelling external dysfunctional strategies, such as pathological online gambling [15,19,21]. However, this indirect effect was moderated by the cohesive family functioning in such a way that the effect of dissociation on problematic online gambling became non-significant at average or high levels of cohesion. The adaptive and supportive family system, therefore, may represent a protective environmental resource, able to partially compensate for personal deficiencies and limit their outcome in pathological gambling [49,70]. These data further support the need to analyse problematic gambling, taking into consideration different levels, supporting the importance of attention that is not only focused on individual characteristics but also on which gamblers consider the latter in their interactions with external and contextual factors [6].

The results of this study should be considered in light of some limitations. First, the cross-sectional design of the research requires prudence in interpreting the causal links in the relationship between the variables taken into account. In future research, the longitudinal approach may offer an opportunity to better clarify the direction of the observed associations. Furthermore, the use of a convenience sampling procedure, the small sample size, and the prevalence of male respondents (albeit this is in line with the greater prevalence of gambling among men, e.g., [71]) may limit the generalizability of the study results. Future research could overcome this issue through the recruitment of larger and more homogeneous samples, with respect to gender, with more adequate sampling procedures. Finally, the possible differences based on the gambling activity have not been considered. The different features of gambling types (such as strategic or luck games) can recreate peculiar experiences that may be preferred based on the type of experience sought by the gambler [68,72]. Such investigation could be an interesting challenge for future research.

## 5. Conclusions

The internet offers the opportunity to engage in numerous online leisure activities, but, for some vulnerable individuals, these can become more than just entertainment and can lead to problematic behaviours (e.g., [6,73]). The present research identified several factors that may be associated with pathological gambling among online gamblers. Specifically, higher levels of alexithymia and dissociation have been identified as personal risk factors for online gambling problems, while cohesive family functioning has proven to be an important protective environmental variable. Such data may have great clinical relevance, suggesting that alexithymia and dissociation should be considered in the clinical assessment of the risk of pathological gambling behaviour. Indeed, both emotional dysregulation and high levels of dissociation have frequently been associated with psychopathology and lower mental resources [74,75,76,77,78], and they could be the focus of interventions aimed at reducing both the direct and indirect effects of alexithymia on problematic online gambling [79,80]. At the same time, the results also highlighted the importance of cohesion in this indirect path. Such finding supports the involvement of contextual and, more specifically, family factors in the preventive activities in at-risk subjects for gambling problems, in line with what has already been previously highlighted in the clinical treatment practice of land-based pathological gambling [81].

## Figures and Tables

**Table 1:** Demographic characteristics of the sample (N = 193).

Characteristics		*M* ± *SD*	*n*	%
*Age* (years)		28.8 ± 10.59		
*Sex*				
	Females		33	17.1
	Males		160	82.9
*Marital Status*				
	Single		152	78.8
	Married		20	10.4
	Cohabiting		17	8.8
	Separated		1	.5
	Divorced		2	1.0
	Widowed		1	.5
*Education*				
	Middle School Diploma		19	9.8
	High School Diploma		106	54.9
	University Degree		48	24.9
	Master’s Degree		10	5.2
	Post-Lauream Specialization		10	5.2
*Occupation*				
	Student		42	21.8
	Working Student		43	22.3
	Employee		73	37.8
	Freelance		5	2.6
	Entrepreneur		2	1.0
	Artisan		13	6.7
	Unemployed		14	7.3
	Retired		1	.5
*Severity of gambling-related problems (SOGS)*				
	Absence Of Gambling Disease		110	57.0
	At Risk For Gambling Disease		33	17.1
	Problematic Gambling		50	25.9

**Table 2:** Correlations, means and standard deviations of the variables.

	1	2	3	4	5	6	7	8	9
1. SOGS	1								
2. DES-II	0.330 **	1							
3. TAS20	0.284 **	0.351 **	1						
4. FACES-IV (1)	−0.241 **	−0.323 **	−0.141	1					
5. FACES-IV (2)	−0.130	−0.186 **	−0.059	0.839 **	1				
6. FACES-IV (3)	0.115	0.347 **	0.265 **	−0.199 **	−0.079	1			
7. FACES-IV (4)	0.180 *	0.359 **	0.303 **	−0.006	0.105	0.445 **	1		
8. FACES-IV (5)	−0.025	0.174 *	0.290 **	0.374 **	0.505 **	0.398 **	0.536 **	1	
9. FACES-IV (6)	0.087	0.304 **	0.248 **	−0.024	0.095	0.607 **	0.512 **	0.377 **	1
*M*	3.306	33.811	49.181	22.72	21.813	17.725	15.399	18.197	16.798
*SD*	4.217	15.483	11.325	6.589	5.813	5.191	4.688	4.664	4.554

**Note:** Bold values indicate significant *p*-values. **. Correlation is significant at the 0.01 level (two-tailed). *. Correlation is significant at the 0.05 level (two-tailed). SOGS = South Oaks Gambling Screen; DESS-II = Dissociative Experience Scale-II; TAS20 = Twenty-Items Toronto Alexithymia Scale; FACES-IV (1) = Cohesion (Family Adaptability and Cohesion Evaluation Scales-IV); FACES-IV (2) = Flexibility (Family Adaptability and Cohesion Evaluation Scales-IV); FACES-IV (3) = Disengaged (Family Adaptability and Cohesion Evaluation Scales-IV); FACES-IV (4) = Enmeshed (Family Adaptability and Cohesion Evaluation Scales-IV); FACES-IV (5) = Rigid (Family Adaptability and Cohesion Evaluation Scales-IV); FACES-IV (6) = Chaotic (Family Adaptability and Cohesion Evaluation Scales-IV).

**Table 3:** Means, standard deviation and comparisons of the types of family functioning based on the levels of gambling disease.

	Absence		At Risk		Problematic		*F*	*p*	*η_p_^2^*	Scheffé Post Hoc
	*M*	*SD*	*M*	*SD*	*M*	*SD*				
**(N = 110)**		**(N = 33)**		**(N = 50)**	
Cohesion	24.227	6.010	21.000	6.955	20.540	6.801	7.171	**<0.001**	0.070	P < A, R
Flexibility	22.645	5.734	20.152	6.893	21.080	4.927	2.931	0.056	0.030	-
Disengaged	17.245	4.530	16.879	5.754	19.340	5.889	3.411	0.035	0.035	-
Enmeshed	14.736	4.323	15.424	4.596	16.840	5.258	3.553	0.031	0.036	-
Rigid	18.255	4.069	17.939	5.645	18.240	5.247	0.060	0.942	0.001	-
Chaotic	16.536	3.960	15.848	4.487	18.000	5.566	2.687	0.071	0.028	-

**Note:** Bold values indicate *p* within the criteria of significance (Bonferroni-adjusted *p* < 0.008); P = problematic gambling; R = at risk for gambling disease; A = absence of gambling disease.

**Table 4:** Means, standard deviation and comparisons of the alexithymia and dissociation scores based on the levels of gambling disease.

	Absence		At Risk		Problematic		*F*	*p*	Scheffé Post Hoc
	(N = 110)		(N = 33)		(N = 50)				
	*M*	*SD*	*M*	SD	*M*	*SD*			
Alexithyima	47.036	10.932	47.182	10.463	55.220	10.725	10.549	**<0.001**	P > A, R
Dissociation	30.497	13.916	33.636	14.133	41.224	17.242	8.935	**<0.001**	P > A

**Note:** Bold values indicate *p* within the criteria of significance; P = problematic gambling; R = at risk for gambling disease; A = absence of gambling disease.

**Table 5:** Coefficients of the model.

Antecedent	Consequent										
	M						Y				
		Coeff.	SE	*p*	95% CI		Coeff.	SE	*p*	95% CI	Test(s) of Highest Order Unconditional Interaction(s)
X	*a*	0.048	0.099	<0.001	[0.2971; 0.6625]	*c′*	0.083	0.027	0.002	[0.0306; 0.1356]	
M		-	-	-	-	*b_1_*	0.206	0.060	<0.001	[0.0885; 0.3238]	
W		-	-	-	-	*b_2_*	0.148	0.102	0.148	[−0.0532; 0.3496]	
M × W		-	-	-	-	*b_3_*	0.007	0.003	0.010	[−0.0128; −0.0018]	Δ*R^2^* = 0.030 *F* (1, 188) = 6.861, *p* < 0.01
constant	*i_M_*	1.021	0.467	0.031	[0.9920; 19.4307]	*i_Y_*	−5.765	2.814	0.042	[−11.3152; −0.2144]	
	*R^2^ =* 0.123 *F* (1, 191) = 26.838, *p <* 0.001					*R^2^* = 0.190 *F*(4, 188) = 10.987, *p* < 0.001					

**Note:** X = alexithymia; M = dissociation; W = cohesion; Y = problematic gambling.

## Data Availability

The data presented in this study are available on request from the corresponding author. The data are not publicly available, for reasons of privacy.
